# Mitochondrial dynamics: quantifying mitochondrial fusion *in vitro*

**DOI:** 10.1186/1741-7007-8-99

**Published:** 2010-07-26

**Authors:** Alexis Jourdain, Jean-Claude Martinou

**Affiliations:** 1 Department of Cell Biology, University of Geneva, Quai Ernest-Ansermet 30, 1211 Geneva 4, Switzerland

## Abstract

Mitochondrial fusion is an essential process for preserving the integrity and stability of mitochondrial DNA; however, regulation of this process remains largely mysterious. In this issue of *BMC Biology*, Schauss and colleagues describe a simple, reliable, and robust novel assay that allows fusion of mammalian mitochondria to be quantified *in vitro*.

See research article: http://biomedcentral.com/1741-7007/8/100

## 

Mitochondria are mainly known as the 'power house' of eukaryotic cells because they are able to catalyze the production of ATP through oxidative phosphorylation. However, these organelles are not restricted to this unique function but fulfill a number of other tasks, including regulation of calcium homeostasis and amino acid metabolism or the citric acid and the urea cycles, and as such participate actively in life and death of cells. Mitochondria contain their own genome that is packaged into nucleoid-like structures containing several mitochondrial DNA (mtDNA) molecules. In humans, each mtDNA molecule encodes 13 proteins, 2 ribosomal RNAs and 22 tRNAs. To function correctly, mitochondria need to be dynamic: they move, fragment and fuse continuously. On one hand, fragmentation or fission is necessary to produce new mitochondria from a 'mother mitochondrion' or to isolate and target damaged parts of one mitochondrion for degradation by mitophagy [1]. On the other hand, fusion allows the mixing of matrix contents of different mitochondria, including their genetic information. Impairment of mitochondrial fusion leads to accumulation of mutations in the mitochondrial genome and finally to loss of mtDNA molecules by a mechanism that is still unclear [2]. Consequently, all mtDNA-encoded proteins, which are core subunits of the respiratory chain, are downregulated and oxidative phosphorylation is impaired, leading to cell dysfunctions. Thus, loss of mtDNA integrity and stability could be the cause of several neurodegenerative disorders that have been associated with mitochondrial fusion impairment, including the inherited diseases Charcot-Marie-Tooth type IIA and optic nerve atrophy. It is therefore important to unravel the principles of mitochondrial fusion by identifying all the components that constitute the core fusion machinery and to understand better how this machinery is controlled and integrated into cell signaling pathways. In addition, it would be useful to identify chemical compounds that could modify mitochondrial dynamics for research or therapeutic use. Until now, one of the limitations in the research on mitochondrial dynamics, especially in mammals, has been the lack of a precise and reliable assay to quantify mitochondrial fusion and fission. An important step towards this goal has now been accomplished by Schauss and colleagues, who have set up an elegant assay allowing quantification of mitochondrial fusion *in vitro *[3].

## Some of the key regulators of mitochondrial fusion are known

Mitochondrial fusion requires the coordinated fusion of the outer and inner membranes. The whole process relies largely on dynamin-like proteins that hydrolyze GTP [4]. For fusion, mitochondria have mitofusins (Fzo1 in yeast) on the surface of their outer membranes [5]. These molecules allow tethering of two organelles before fusion of the outer membrane itself occurs. Lipid mixing of the outer membrane could be catalyzed by lipid-modifying enzymes, such as mitochondrial phospholipase D (mito-PLD) [6]. The mechanism of mitochondrial inner membrane fusion is less clear. It has been demonstrated, however, that it largely depends on another dynamin-like GTPase, Opa1 (Mgm1 in yeast) [7]. It is not known how fusion of inner and outer membranes is coordinated in mammals, but in yeast a third protein of the outer membrane, Ugo1, which interacts with both Fzo1 and Mgm1, may fulfill the role of a membrane fusion coordinator [8]. Mitochondrial fission relies on the cytosolic dynamin-related protein 1 (Drp1 in mammals, Dnm1 in yeast), which uses the protein Fis1 as a receptor on the mitochondrial outer membrane [9]. Mitochondrial dynamins can be regulated by post-translational modifications, including phosphorylation, sumoylation and ubiquitination [9,10], which impact on their function and consequently on mitochondrial shape and dynamics.

Our knowledge of how mitochondria fuse and fragment has significantly increased over the past decade, mainly thanks to genetic studies performed in *Drosophila *or yeast that allowed identification of key players of these processes. However, the picture is incomplete and additional components of the fusion and fission machineries certainly remain to be identified. Moreover, the intracellular cascades that control these machineries are not well characterized yet.

In 2004, Jody Nunnari and colleagues [11] were able to induce, for the first time, fusion of isolated mitochondria *in vitro*. Mitochondria of yeast expressing either mitochondrially targeted GFP or dsRed were isolated, mixed, centrifuged at 4°C to promote membrane tethering, and resuspended at 37°C. Under these conditions, mitochondrial fusion could be observed by confocal or electron microscopy. This cell-free fusion reaction confirmed the requirement of GTP, ATP, an intact membrane potential, and Fzo1 and Mgm1 for fusion of the outer and inner mitochondrial membranes, respectively, as shown in previous cell fusion assays [12]. However, although useful, this cell-free assay is not optimal to obtain a reliable quantification of mitochondrial fusion, in part because the merge of green and red fluorescent markers is only semi-quantitative. Moreover, the high resolution required to image fused mitochondria with a confocal microscope is difficult to combine with automation and high-throughput screening.

## A new, sensitive, and highly adaptable mitochondrial fusion assay

**Figure 1:**
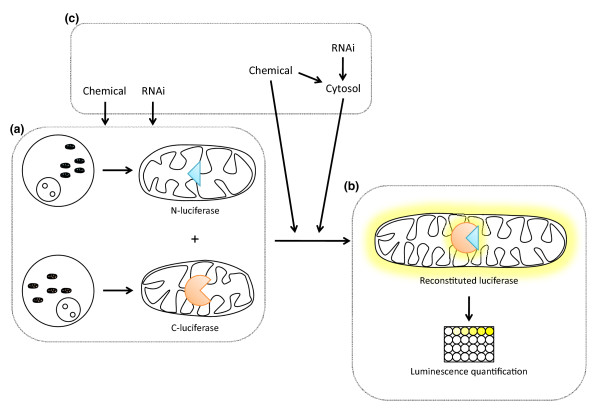
**Identification of new regulators of mitochondrial fusion using a novel quantitative bi-molecular complementation assay**. **(a) **Mitochondria from two cell lines expressing either the amino-terminal part of luciferase or its carboxy-terminal part are isolated. **(b) **Upon mixing of both populations, mitochondrial fusion occurs, leading to the reconstitution of the luciferase into a functional protein. The emission of light is quantified with a plate reader and is proportional to the amount of mitochondrial fusion. **(c) **Several parameters of the assay can be modified. First, one or both of the cell lines from which mitochondria are isolated can be pre-treated - for example, with chemicals (for example, forskolin) or RNA interference (RNAi; for example, PKA). Then, cytosol from different sources can be added to the fusion mixture. At the same time, different chemicals can be included in the mixture, which gives rise to the possibility to perform high throughput screens for new modulators of mitochondrial dynamics.

The novel assay described by Heidi McBride and colleagues [3] has overcome these limitations and has been applied to mammalian mitochondria. These authors targeted the amino- or carboxy-terminal part of *Renilla *luciferase to the mitochondrial matrix of two distinct human cell lines. In addition, both proteins were fused to a leucine zipper to ensure their dimerization. The principle is that upon fusion of mitochondria, the two halves of luciferase are reconstituted to a functional protein, able to emit light in the presence of coelenterazine (Figure 1). The fusion protocol, in particular the centrifugation step to promote tethering of mitochondria, is largely inspired by that described by Meeusen *et al. *[11]. The assay turned out to be very accurate, with an impressive signal-to-noise ratio, which is essential, for example, for high-throughput screening. Using this acellular assay, the authors confirmed previous data from Meeusen *et al*. that suggested that, for mitochondria to be fusogenic *in vitro*, they need energy and inner membrane potential, whereas the presence of cytosol is dispensable. However, they observed that in the presence of cytosol, mitochondrial fusion was modulated, either positively or negatively depending on either the source of cytosol or its state of activation at the time of its extraction. For example, addition of cytosol from cells in which the PKA/cAMP signaling pathway had been activated before extraction led to stimulation of the core mitochondrial fusion machinery, as predicted by *in cellulo *data. Thus, the authors were able to reproduce, in a test tube, cytosolic signaling cascades leading to quantifiable mitochondrial fusion.

## Future directions

These data suggest that this assay could be used as a reliable readout to identify new factors that are part of, or control, the core mitochondrial fusion machinery. Differences in the activities of the cytosols from multiple sources suggest a tissue specificity of the factors regulating mitochondrial fusion. These factors could be purified by classical biochemical procedures, their specific activities being measured by *Renilla *luciferase activities in the mitochondrial fusion assay. Moreover, high throughput technologies can be envisaged, in particular screening of large libraries of chemicals. At the moment, only mitochondria from cell lines expressing the split luciferases are available. This may restrict the number of assays performed and may be a limitation for high throughput screening. This limitation could be overcome by expressing these luciferase reporters in transgenic mice and by isolating mitochondria from different tissues of these animals. This would also allow testing whether different mitochondria express various forms of the core fusion machinery, which may respond differently to the signaling cascades. In conclusion, this novel assay should be useful to those who more and more are interested in quantifying mitochondrial fusion and should boost research aiming at understanding the mechanisms that govern mitochondrial dynamics. Ultimately, this could lead to better understanding and treatment of mitochondrial diseases in humans.
